# One-Year Renal Recovery Following Acute Kidney Injury in Stroke Patients: A Prospective Longitudinal Follow-Up Study From Hayatabad Medical Complex Peshawar

**DOI:** 10.7759/cureus.100592

**Published:** 2026-01-01

**Authors:** Imran Khan, Mehwash Iftikhar, Ameer Hamza, Ayesha Jamal, Muhammad Numan Saleem, Sheraz J Khan

**Affiliations:** 1 Department of Medicine, Hayatabad Medical Complex Peshawar, Peshawar, PAK; 2 Department of Family Medicine, St. Boniface Hospital, Winnipeg, CAN; 3 Department of Internal Medicine, Hayatabad Medical Complex Peshawar, Peshawar, PAK

**Keywords:** acute kidney injury recovery, longitudinal study, pakistan, renal function, stroke outcomes

## Abstract

Background and aim

Acute kidney injury (AKI) significantly affects stroke patients, yet long-term recovery patterns remain poorly understood, especially in resource-limited settings. The incidence of AKI in stroke patients is notable, particularly in severe cases, but it is unclear whether these injuries are permanent or reversible; a critical knowledge gap for patient counseling, resource allocation, and healthcare planning where dialysis is limited. This prospective longitudinal follow-up study assessed one-year renal recovery outcomes in stroke-associated AKI, examining recovery patterns, temporal trajectories, and influencing factors.

Methods

This prospective longitudinal follow-up study tracked 33 stroke patients who developed AKI from an original cohort of 214 patients over 12 months (August 2023 to August 2024). Renal function was assessed every three months using serum creatinine and estimated glomerular filtration rate (eGFR). Complete recovery was defined as a return to baseline creatinine ±0.2 mg/dL or an eGFR >60 mL/min/1.73 m².

Results

Of the 33 patients with AKI, 31 (93.9%) completed the 12-month follow-up. Among survivors, 24/31 (77.4%) achieved complete renal recovery without intervention, while 7/31 (22.6%) had persistent mild dysfunction. Two patients died at three months with persistent dysfunction, yielding a population-level recovery rate of 72.7% (24/33) when accounting for early mortality. No patients required dialysis per the Kidney Disease: Improving Global Outcomes (KDIGO) guidelines. Patients with hemorrhagic stroke showed recovery rates comparable to those with ischemic stroke (83.3% vs. 69.2%, p = 0.543). Patients with severe stroke had slower recovery (median 8.2 months vs. 5.1 months for moderate-to-severe cases, p = 0.041). Predictors of faster recovery included lower peak creatinine (HR, 2.63 per mg/dL decrease; 95% CI, 1.31-5.26; p = 0.006), moderate-to-severe versus severe stroke (HR, 1.89; 95% CI, 1.12-3.18; p = 0.017), and younger age (HR, 1.12 per year decrease; 95% CI, 1.02-1.23; p = 0.021).

Conclusions

Most stroke-related AKI demonstrates excellent spontaneous recovery within one year, supporting conservative management approaches in similar patients. Even patients with severe strokes and multiple comorbidities showed robust long-term renal recovery in Pakistani healthcare settings. However, these findings should be confirmed in larger multicenter studies, given the small sample size and potential survival bias due to early mortality.

## Introduction

Acute kidney injury (AKI) represents a sudden deterioration in kidney function, measured through changes in blood creatinine levels or estimated glomerular filtration rate (eGFR). AKI commonly affects hospitalized individuals, with an incidence of 23.4% based on Kidney Disease: Improving Global Outcomes (KDIGO) guidelines. This condition is associated with an increased risk of mortality across various clinical scenarios. In developing nations such as Pakistan, patients with AKI face significant challenges related to healthcare costs and access to renal replacement therapy [1].

Cerebrovascular events, classified as acute ischemic stroke or intracranial hemorrhage, represent a substantial global health burden. Stroke is the second leading cause of death worldwide and the fourth leading cause of disability. While precise epidemiological data from Pakistan remain limited, estimates suggest approximately 350,000 new stroke cases annually, with a prevalence of 4.8% and mortality rates ranging from 11% to 30% [1].

In our previous study conducted at Hayatabad Medical Complex Peshawar, from February to July 2023, we investigated the frequency of AKI among patients presenting with acute stroke. Among 214 stroke patients, AKI occurred in 33 (15.4%). Notably, AKI was observed in all patients with severe stroke (26/26, 100%) but in none of those with mild or moderate stroke. The study concluded that stroke type and severity were stronger predictors of AKI development than comorbidities [1]. However, that analysis focused exclusively on the acute hospitalization phase and could not determine whether the high AKI rates observed would lead to permanent renal impairment requiring long-term dialysis or represent potentially reversible acute injuries.

Contemporary evidence suggests that the timing and pattern of renal recovery may be more predictive of long-term outcomes than initial AKI severity alone. This evolving paradigm emphasizes recovery trajectories rather than severity staging, which is particularly relevant for stroke-associated AKI, where neurological factors may influence recovery dynamics [2].

The intersection of stroke and kidney dysfunction involves complex bidirectional organ crosstalk mediated through multiple pathways. Husain-Syed et al. demonstrated that approximately 20% of patients with acute brain injury develop AKI through mechanisms including (1) neurogenic stunned myocardium resulting in reduced cardiac output and renal hypoperfusion; (2) sympathoadrenergic hyperactivation leading to renal vasoconstriction and altered autoregulation; (3) activation of the renin-angiotensin-aldosterone system; and (4) systemic inflammatory responses with cytokine release contributing to both blood-brain barrier disruption and renal tubular injury [3].

Biomarker research has further elucidated mechanisms underlying AKI recovery. In a longitudinal study of 656 patients with AKI, Wen et al. demonstrated that sustained injury markers, including urinary interleukin-18, kidney injury molecule-1, and neutrophil gelatinase-associated lipocalin, were associated with a two- to threefold increased risk of chronic kidney disease (CKD), whereas protective markers, such as urinary uromodulin and epidermal growth factor, were associated with a 40% reduced risk of CKD [4].

Recent studies have highlighted important knowledge gaps in outcomes related to stroke-associated AKI. In the Third China National Stroke Registry, Zhou et al. reported that acute kidney disease occurred in 3.9%-9.9% of patients with ischemic stroke and was strongly associated with increased all-cause mortality and post-stroke disability [5]. Similarly, a Pakistani study by Shahzadi et al. reported an AKI incidence of 19.2%, with a highly significant association with 30-day mortality [6]. Like our original research, these studies primarily focused on short-term outcomes during hospitalization or within 30 days of stroke onset, leaving long-term renal recovery patterns insufficiently explored.

Building on our original findings, this prospective longitudinal follow-up study aims to address this critical knowledge gap by evaluating long-term renal recovery following stroke-associated AKI in a Pakistani healthcare setting. Specifically, we sought to determine the proportion of patients achieving complete renal recovery at 12 months post-stroke, characterize temporal recovery patterns, identify factors associated with recovery time, assess the need for long-term renal replacement therapy, and examine the relationship between renal recovery and functional outcomes. We hypothesized that most patients would achieve renal recovery without requiring chronic dialysis, with recovery patterns influenced by stroke severity, stroke type, and baseline patient characteristics [1].

This study represents the first 12-month longitudinal follow-up of stroke-associated AKI in Pakistan and South Asia. Our findings demonstrate that most patients experience complete renal recovery without the need for dialysis. Importantly, we show that recovery kinetics differ by stroke severity, while ultimate recovery potential remains comparable across stroke types and severity levels. These results directly address a critical gap in understanding long-term outcomes in this high-risk population.

## Materials and methods

Study design and setting

This prospective longitudinal follow-up study tracked all 33 patients from our original cohort who developed AKI following stroke. The study was conducted at Hayatabad Medical Complex, Peshawar, Pakistan, from August 2023 to August 2024, representing a 12-month follow-up period after completion of the initial study (February to July 2023). The last patient enrolled in July 2023 completed a 12-month follow-up in July 2024.

Ethical approval

The study received ethical approval from the Institutional Review Board, Hayatabad Medical Complex (approval no. 2209, dated July 10, 2023). All participants provided written informed consent for long-term follow-up. The study was conducted in accordance with the Declaration of Helsinki and local research guidelines.

AKI definition and staging

AKI was defined in the original study according to KDIGO serum creatinine criteria: an increase of ≥0.3 mg/dL within 48 hours or an increase of ≥1.5 times baseline within seven days [7]. KDIGO staging was applied as follows: stage 1 (1.5-1.9 × baseline), stage 2 (2.0-2.9 × baseline), and stage 3 (≥3.0 × baseline or initiation of renal replacement therapy). Urine output criteria (<0.5 mL/kg/h for six to 12 hours) were not systematically recorded because of practical limitations in ward-level monitoring, representing a study limitation. Baseline creatinine was defined as the most recent outpatient value within three months prior to stroke or, if unavailable, the lowest value recorded during hospitalization, in accordance with KDIGO recommendations.

Participant selection and follow-up

Of the 33 patients who developed AKI in the original study, all were eligible for longitudinal follow-up. These patients included 18 with hemorrhagic stroke and 15 with ischemic stroke, all of whom had severe strokes (National Institutes of Health Stroke Scale (NIHSS) >20) [8] or moderate-to-severe strokes (NIHSS 16-20) in the original cohort. Inclusion criteria for continued participation included survival beyond the initial hospitalization, residence within 100 km of Peshawar to allow feasible follow-up, and provision of informed consent for extended monitoring. The 100-km radius was selected based on an assessment of patient travel capacity and coverage by the community health worker network, balancing inclusivity with practical follow-up completion. Exclusion criteria included initiation of chronic dialysis during the index hospitalization, development of end-stage renal disease from other causes, or loss to follow-up, defined as missing two consecutive scheduled visits (a six-month gap).

Clinical management protocol

All patients received standardized conservative management during hospitalization and follow-up, including (1) fluid management with isotonic saline for volume repletion while avoiding nephrotoxic contrast when possible; (2) nephrotoxin avoidance, including discontinuation or dose adjustment of nonsteroidal anti-inflammatory drugs, aminoglycosides, and angiotensin-converting enzyme inhibitors during the acute phase; (3) blood pressure management, targeting a systolic pressure of 140-180 mmHg during acute stroke in accordance with guidelines, with individualized targets during recovery; (4) monitoring, consisting of daily serum creatinine measurements during hospitalization and assessments every three months during follow-up; and (5) nephrology referral for persistent eGFR <30 mL/min/1.73 m², uremic symptoms, or refractory fluid overload. Dialysis was considered only for standard KDIGO indications, including severe uremia, refractory hyperkalemia (K >6.5 mEq/L), volume overload unresponsive to diuretics, or metabolic acidosis (pH <7.1).

Data collection protocol

Patients were evaluated every three months (at three, six, nine, and 12 months post-discharge) using a standardized protocol. Primary outcome measures included serum creatinine and eGFR, calculated using the Chronic Kidney Disease Epidemiology Collaboration (CKD-EPI) equation [9]. Secondary measures included blood pressure control, functional status assessed using the modified Rankin Scale (mRS) [10], and adverse events, including hospitalization for any cause, cardiovascular events (myocardial infarction, heart failure, or arrhythmias), progression to CKD, need for renal replacement therapy, and mortality. For patients unable to attend hospital visits, community health workers collected blood samples and performed basic assessments. Laboratory investigations were conducted using the same equipment and protocols as in the original study to ensure consistency.

Survival and recovery analysis approach

Recovery analyses were performed in patients who survived and completed follow-up assessments. Patients who died during the follow-up period were analyzed separately as early mortality cases. Recovery rates are reported both as percentages of patients completing follow-up (for clinical recovery assessment) and as percentages of the original cohort (for population-level outcomes that account for mortality). This dual reporting approach provides clinically relevant recovery data while accounting for the competing risk of death.

Recovery definitions

Complete recovery was defined as a return to baseline creatinine ±0.2 mg/dL or achievement of an eGFR >60 mL/min/1.73 m² sustained over two consecutive visits. The ±0.2 mg/dL margin accounts for normal biological variability in creatinine measurements and is consistent with KDIGO recovery criteria. Partial recovery was defined as improvement to within 25% of baseline creatinine values or an eGFR of 45-60 mL/min/1.73 m². Non-recovery was defined as persistent elevation of creatinine >25% above baseline or an eGFR <45 mL/min/1.73 m² at 12 months.

Statistical analysis

Data were analyzed using IBM SPSS Statistics for Windows, Version 25.0 (Released 2017; IBM Corp., Armonk, NY, USA). Time-to-recovery analysis was performed using Kaplan-Meier survival curves with log-rank tests for group comparisons. Proportional hazards assumptions were verified using Schoenfeld residuals (all p > 0.05). Cox proportional hazards models were used to identify predictors of recovery time. Given the limited sample size (31 patients and 24 recovery events), univariate Cox regression was first performed for all variables. Only variables with p < 0.20 in univariate analysis were included in the multivariate model to reduce the risk of overfitting. The final multivariate model included age, peak creatinine, and stroke severity. HRs <1.0 indicate slower recovery (longer time to achieve complete recovery), whereas HRs >1.0 indicate faster recovery. Cox regression results should be interpreted cautiously because of the limited sample size relative to the number of covariates, and findings require validation in larger cohorts. Repeated-measures analysis of variance was used to assess longitudinal changes in renal function parameters. Missing data (<5%) were handled using the last observation carried forward method. Statistical significance was set at p < 0.05.

## Results

Baseline characteristics and follow-up completion

Of the 33 patients with AKI from the original cohort, 31 (93.9%) completed the 12-month follow-up period. Two patients died three months post-discharge. The completing cohort had a mean age of 58.1 ± 6.5 years, with 20 patients (64.5%) being male. Severe strokes (NIHSS >20) were present in 24 patients (77.4%), while seven patients (22.6%) had moderate-to-severe strokes (NIHSS 16-20). Baseline characteristics of the follow-up cohort were consistent with those reported in the original study, with hemorrhagic strokes comprising 58.1% (n = 18) and ischemic strokes 41.9% (n = 13).

Recovery patterns and timeline

At three months post-discharge, nine of 31 patients (29.0%) had achieved target creatinine or eGFR values at that single visit (provisional recovery). Recovery increased progressively over time, with 16 of 31 patients (51.6%) at six months, 21 of 31 (67.7%) at nine months, and 24 of 31 (77.4%) achieving complete sustained recovery at 12 months. Seven patients (22.6% of survivors) had persistent mild kidney dysfunction at 12 months (eGFR 50-59 mL/min/1.73 m²), meeting criteria for CKD stage 3a; however, most lacked proteinuria or other markers of structural kidney damage, suggesting incomplete recovery rather than progressive disease.

Two patients (6.1% of the original cohort) died three months post-discharge. At the time of death, both had persistent renal dysfunction (eGFR <60 mL/min/1.73 m²). When accounting for early mortality in population-level outcomes, the complete recovery rate was 72.7% (24 of 33) in the original AKI cohort (Table 1).

**Table 1:** Recovery rates by time point Percentages were calculated among the 31 patients who completed the 12-month follow-up. Two patients (6.1% of the original cohort, n = 33) died at three months with persistent renal dysfunction (eGFR <60 mL/min/1.73 m²) and were not included in subsequent time points. Population-level complete recovery, accounting for early mortality, was 72.7% (24 of 33) of the original AKI cohort. AKI, acute kidney injury; eGFR, estimated glomerular filtration rate

Time point	Complete recovery, n (%)	Partial recovery, n (%)	No recovery, n (%)
Three months	9 (29.0)	15 (48.4)	7 (22.6)
Six months	16 (51.6)	11 (35.5)	4 (12.9)
Nine months	21 (67.7)	7 (22.6)	3 (9.7)
12 months	24 (77.4)	5 (16.1)	2 (6.5)

Time-to-recovery analysis

**Figure 1:**
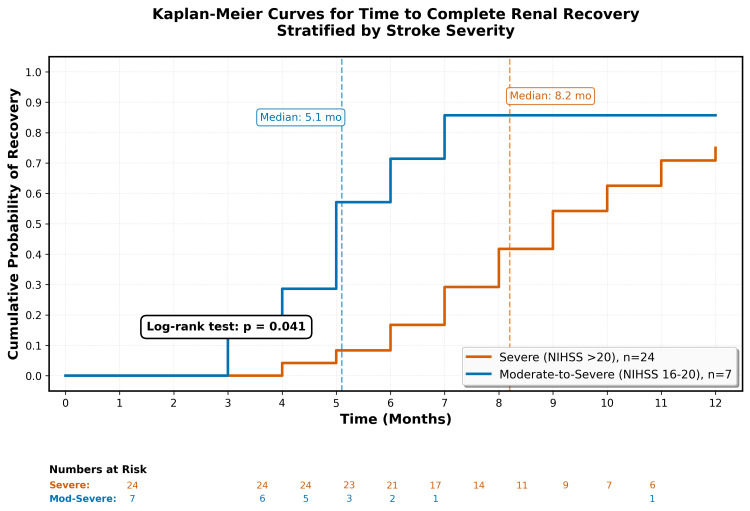
Kaplan-Meier curves for time to complete renal recovery NIHSS, National Institutes of Health Stroke Scale

Kaplan-Meier analysis demonstrated that stroke severity significantly influenced recovery time. Patients with severe strokes (NIHSS >20) had a median time to complete recovery of 8.2 months (95% CI: 6.8-9.6), compared with 5.1 months (95% CI: 3.9-6.3) for patients with moderate-to-severe strokes (log-rank p = 0.041). The cumulative probability of recovery increased steadily over time in both groups, with the severe stroke group demonstrating slower but ultimately comparable recovery rates relative to the moderate-to-severe group (Figure 1).

Creatinine trends over time

Mean creatinine levels steadily improved from peak AKI values documented in the original study. The mean baseline creatinine in the original cohort was 0.98 ± 0.24 mg/dL, with peak levels of 1.42 ± 0.38 mg/dL during the acute AKI phase. During follow-up, mean creatinine values were 1.38 ± 0.35 mg/dL at discharge, decreasing to 1.22 ± 0.28 mg/dL at three months, 1.09 ± 0.21 mg/dL at six months, 1.04 ± 0.18 mg/dL at nine months, and 1.01 ± 0.16 mg/dL at 12 months. Among patients achieving complete recovery, final creatinine values at 12 months (mean 0.97 ± 0.14 mg/dL) were statistically indistinguishable from pre-stroke baseline values (mean 0.98 ± 0.24 mg/dL, p = 0.782 by paired t-test), indicating true restoration of renal function rather than adaptation to a lower functional baseline, which would represent residual impairment.

Impact of stroke type and severity on recovery

Consistent with findings from the original study that hemorrhagic strokes had higher AKI incidence (26.9%) compared to ischemic strokes (10.2%), we examined whether stroke type influenced recovery patterns. Patients with hemorrhagic stroke (n = 18) showed comparable recovery to those with ischemic stroke (n = 13). At 12 months, complete recovery was achieved in 15/18 (83.3%) hemorrhagic stroke patients versus 9/13 (69.2%) ischemic stroke patients (p = 0.543), suggesting that although hemorrhagic strokes were more likely to cause AKI initially, they did not adversely affect long-term recovery potential.

In contrast, stroke severity significantly influenced recovery time. Patients with severe strokes (NIHSS >20) had a median time to complete recovery of 8.2 months (95% CI: 6.8-9.6) compared to 5.1 months (95% CI: 3.9-6.3) for moderate-to-severe strokes (p = 0.041) (Table 2).

**Table 2:** Recovery characteristics by stroke type and severity Left columns compare recovery by stroke type (hemorrhagic vs. ischemic), and right columns compare recovery by stroke severity (severe vs. moderate to severe). Recovery rates represent complete renal recovery (return to baseline creatinine ±0.2 mg/dL or eGFR >60 mL/min/1.73 m²) among patients who completed follow-up (n = 31). eGFR, estimated glomerular filtration rate; NIHSS, National Institutes of Health Stroke Scale

Parameter	Hemorrhagic (n = 18)	Ischemic (n = 13)	p-Value	Severe (NIHSS >20) (n = 24)	Moderate to severe (NIHSS 16-20) (n = 7)	p-Value
Median recovery time, months (95% CI)	7.8 (6.2-9.4)	6.9 (5.1-8.7)	0.421	8.2 (6.8-9.6)	5.1 (3.9-6.3)	0.041
Six-month recovery rate, n (%)	9/18 (50.0%)	7/13 (53.8%)	0.823	10/24 (41.7%)	6/7 (85.7%)	0.034
12-month recovery rate, n (%)	15/18 (83.3%)	9/13 (69.2%)	0.543	18/24 (75.0%)	6/7 (85.7%)	0.567
Final eGFR, mL/min/1.73m² (mean ± SD)	66.8 ± 13.1	69.2 ± 11.4	0.563	66.1 ± 12.8	72.4 ± 10.2	0.243

Dialysis requirements and complications

During the 12-month follow-up, no patients required initiation of dialysis. Two patients had nadir eGFR values between 30 and 44 mL/min/1.73 m² but maintained stable renal function without uremic symptoms or fluid overload requiring intervention. No patients progressed to CKD stage 4 or 5.

Predictors of recovery

Multivariate Cox regression analysis identified factors associated with recovery time. Variables were initially screened using univariate analysis, with those meeting p < 0.20 included in the multivariate model. The final model incorporated age, peak creatinine, and stroke severity. Faster recovery was associated with lower peak creatinine during AKI (HR 0.38 per mg/dL higher, 95% CI: 0.19-0.76, p = 0.006), moderate-to-severe versus severe stroke (HR 1.89, 95% CI: 1.12-3.18, p = 0.017), and younger age (HR 0.89 per year older, 95% CI: 0.81-0.98, p = 0.021). HRs <1.0 indicate slower recovery (longer time to complete recovery), whereas HR >1.0 indicates faster recovery (Table 3).

**Table 3:** Predictors of recovery time (Cox regression analysis) HRs <1.0 indicate slower recovery (longer time to achieve complete recovery), whereas HR >1.0 indicates faster recovery. Results should be interpreted cautiously due to the limited sample size (31 patients and 24 events) relative to the number of covariates included in the model.

Variable	HR (95% CI)	p-Value
Age (per year older)	0.89 (0.81-0.98)	0.021
Peak creatinine (per mg/dL higher)	0.38 (0.19-0.76)	0.006
Severe vs. moderate-to-severe stroke	0.53 (0.31-0.89)	0.017
Hemorrhagic vs. ischemic stroke	0.87 (0.52-1.45)	0.594
Male gender	1.08 (0.65-1.79)	0.766
Baseline creatinine (per mg/dL)	0.92 (0.54-1.57)	0.756

Functional outcomes and quality of life

mRS scores improved significantly over the 12-month follow-up among patients who achieved complete renal recovery. At 12 months, patients with complete recovery had better functional outcomes compared to those with persistent renal dysfunction (mean mRS 2.1 ± 1.2 vs. 3.4 ± 1.1, p = 0.003). No cardiovascular events attributable to renal dysfunction, such as uremic pericarditis, severe hyperkalemia, or volume overload, occurred during the follow-up period.

## Discussion

This 12-month longitudinal follow-up study provides important evidence of excellent long-term renal recovery in stroke-associated AKI within a Pakistani healthcare setting. Among the 31 patients who survived and completed follow-up, 24 (77.4%) achieved complete recovery without requiring dialysis. When accounting for early mortality, the population-level complete recovery rate was 72.7% (24/33). To our knowledge, these findings represent the first detailed longitudinal analysis of stroke-related AKI outcomes in South Asia [1].

Our follow-up study directly addresses the most significant limitation of our original research, which could not determine whether the high AKI rates observed, particularly the universal occurrence in severe stroke patients, would result in permanent renal impairment or reversible injury. The current study provides reassuring evidence that despite the high acute AKI incidence previously documented (15.4% overall, 26.9% in hemorrhagic strokes, and 100% in severe strokes), long-term renal prognosis is favorable [1].

Recovery in severe stroke patients

One of the most striking findings from our original study was that all severe stroke patients (26/26, 100%) developed AKI. Our follow-up data demonstrate that despite this universal occurrence, 75.0% (18/24) of severe stroke patients who survived achieved complete renal recovery at 12 months. While these patients had slower recovery times (median 8.2 months) compared to moderate-to-severe cases (5.1 months, p = 0.041), their final recovery rates were comparable (75.0% vs. 85.7%, p = 0.567), suggesting that stroke severity primarily affects recovery kinetics rather than ultimate recovery potential [1].

Stroke type and recovery outcomes

Our original study identified a significantly higher AKI incidence in hemorrhagic strokes (26.9%) compared to ischemic strokes (10.2%, p < 0.005). The current follow-up demonstrates that despite this differential susceptibility, recovery rates were comparable between stroke types (83.3% in hemorrhagic vs. 69.2% in ischemic, p = 0.543). This dissociation between acute risk and long-term outcomes suggests that the pathophysiological mechanisms triggering AKI in hemorrhagic strokes are largely reversible once the acute phase resolves [1].

Understanding prolonged recovery beyond three to six months

The observation that some patients achieved complete recovery beyond six months merits discussion. This extended recovery trajectory represents prolonged acute kidney disease rather than CKD, as evidenced by eventual normalization of creatinine and eGFR to baseline values without proteinuria or progressive decline. Stroke-associated AKI primarily involves reversible acute tubular injury and neurohormonal disturbances, explaining the capacity for late recovery, unlike the irreversible fibrotic changes characteristic of CKD. The temporal progression of recovery, from 29.0% at three months to 77.4% at 12 months, aligns with current understanding of renal repair mechanisms. The initial rapid recovery phase likely reflects resolution of acute tubular injury, while subsequent improvement reflects adaptive processes and structural remodeling. This pattern supports contemporary approaches emphasizing recovery trajectories over single-point severity assessments [2].

Creatinine normalization as evidence of true recovery

The mean baseline creatinine in our original cohort was 0.98 ± 0.24 mg/dL, rising to peak levels of 1.42 ± 0.38 mg/dL during acute AKI. Recovered patients achieved final creatinine values of 0.97 ± 0.14 mg/dL at 12 months, not significantly different from baseline (p = 0.782). This indicates true restoration of renal function rather than stabilization at reduced levels, supporting the hypothesis that stroke-associated AKI predominantly involves reversible acute tubular injury [1].

Clinical management implications

The absence of dialysis requirements in our cohort should not be interpreted as evidence that dialysis is never needed in stroke-associated AKI. International literature documents that 5-15% of severe stroke patients may require renal replacement therapy. Our findings support conservative management with close monitoring as the initial approach, with dialysis reserved for standard indications (severe uremia, refractory hyperkalemia, volume overload, or metabolic acidosis) per KDIGO guidelines [11]. The key message is that most stroke-AKI patients can be managed conservatively with watchful waiting, but aggressive intervention, including dialysis, should be promptly initiated when indicated. Based on our experience, we recommend (1) daily creatinine monitoring during acute hospitalization; (2) nephrotoxin avoidance (NSAIDs, aminoglycosides, contrast where possible); (3) volume management guided by clinical assessment; (4) blood pressure targets individualized for stroke type; (5) outpatient follow-up at three-month intervals during the first year; (6) nephrology referral for eGFR <30 mL/min/1.73m², uremic symptoms, or lack of recovery trajectory by six months. This approach supports KDIGO 2024 guidelines, emphasizing the avoidance of unnecessary renal replacement therapy [11].

Comorbidities and recovery

Our original study identified hypertension and diabetes as prevalent comorbidities, with 16/31 patients (51.6%) having both conditions, 8/31 (25.8%) having one comorbidity, and 7/31 (22.6%) having neither. While these comorbidities were common, multivariate analysis indicated that stroke type and severity were stronger predictors of AKI development. The current follow-up confirms that this pattern extends to recovery: among patients with both hypertension and diabetes, 12/16 (75.0%) achieved complete recovery compared to 12/15 (80.0%) in patients without both comorbidities (p = 0.732). Similarly, recovery rates did not differ significantly between patients with any comorbidity versus those without (18/24, 75.0% vs. 6/7, 85.7%; p = 0.542). Cox regression analysis showed that neither hypertension (HR 0.92, 95% CI: 0.48-1.76, p = 0.801) nor diabetes (HR 0.85, 95% CI: 0.44-1.64, p = 0.628) significantly influenced recovery time, suggesting that acute stroke-related AKI mechanisms are distinct from chronic comorbidity-related kidney disease [1].

Comparison with international literature

The contrast between our findings and some international studies highlights the importance of population-specific research. Zhou et al.’s analysis showed higher mortality and disability rates associated with acute kidney disease [5], whereas our extended follow-up demonstrates that survivors have excellent recovery potential. Similarly, Shahzadi et al.’s Pakistani study reported strong associations with 30-day mortality [6], but our results indicate that patients who survive the acute period exhibit favorable long-term renal recovery patterns.

Kidney-brain interactions and functional outcomes

The strong association between renal recovery and functional outcomes supports the growing recognition of kidney-brain interactions in stroke recovery. Patients achieving complete renal recovery had significantly better functional outcomes at 12 months (mean mRS 2.1 ± 1.2 vs. 3.4 ± 1.1, p = 0.003), although causality cannot be established from our observational design [12].

Healthcare policy implications

Our findings provide important context for healthcare policy development in resource-limited settings. The excellent recovery rates achieved with basic monitoring protocols suggest that expensive interventions, including early dialysis initiation, may not be necessary for most stroke-AKI patients. This has significant implications for healthcare resource allocation in developing countries where dialysis availability is limited [13].

Mechanistic insights and future biomarker research

Longitudinal biomarker research by Wen et al. provides a mechanistic context for our observations. Their demonstration that sustained injury markers correlate with progression risk, while protective markers indicate better outcomes, suggests that our recovered patients likely achieved resolution of underlying injury processes. Future research incorporating biomarker assessments could provide earlier recovery prediction and guide intervention timing [4]. Recent comprehensive reviews have highlighted the complexity of stroke-induced renal dysfunction mechanisms. Chen et al.’s analysis identified key pathways linking stroke to kidney dysfunction, suggesting that stroke-associated AKI may have distinct recovery trajectories compared to other AKI etiologies [14]. Arnold et al.’s meta-analysis demonstrated that AKI following stroke significantly increases mortality risk [15], but our findings suggest that survival beyond the initial period is associated with excellent recovery potential. Fandler-Höfler et al.’s analysis of stroke thrombectomy patients found that AKI was associated with unfavorable three-month outcomes [16], but our broader stroke cohort suggests that fundamental recovery principles may be consistent across different management approaches.

Study limitations

Several study limitations warrant consideration. The relatively small sample size (n = 31 completing follow-up) limits statistical power for complex analyses, and the Cox regression results should be interpreted cautiously. The single-center design may limit generalizability, although our patient demographics appear consistent with broader Pakistani stroke populations. Exclusion of patients with preexisting CKD may have selected for better recovery potential. Importantly, our recovery rates may represent optimistic estimates due to survival bias. Of the original 33 AKI patients, 2/33 (6.1%) died at three months post-discharge before completing recovery assessment; both had persistent renal dysfunction at the time of death (eGFR <60 mL/min/1.73m²), suggesting they would not have achieved recovery if they had survived. Among the 31/33 (93.9%) patients who survived and completed follow-up, 24/31 (77.4%) achieved complete recovery. When accounting for early mortality in population-level outcomes, the complete recovery rate was 24/33 (72.7%) of the original AKI cohort. An additional 7/31 survivors (22.6%) had persistent mild dysfunction (eGFR 50-59 mL/min/1.73m²), yielding a population-level persistent dysfunction rate of 9/33 (27.3%) [1]. The lack of systematic urine output measurement represents a limitation in AKI ascertainment per the complete KDIGO criteria. Biomarker data (injury and repair markers) were not collected, limiting our ability to identify early predictors of recovery or mechanistic insights.

Future research directions

Future research priorities include larger multicenter studies to validate these findings across different Pakistani healthcare systems. Investigation of interventions that might accelerate recovery in severe stroke patients could be particularly valuable, given the longer recovery times in this subgroup (median 8.2 months). Integration of biomarker studies (injury markers, repair markers, and inflammatory mediators) could enhance understanding of recovery processes and enable earlier targeted interventions. Comparative effectiveness research examining different monitoring protocols and nephrology referral thresholds would inform optimal resource utilization in resource-limited settings.

Clinical counseling and patient management

From a healthcare policy perspective, our findings support the development of structured follow-up protocols for stroke-AKI patients in Pakistani settings, emphasizing conservative management while maintaining appropriate monitoring. Healthcare providers can counsel patients and families that while AKI is common in severe strokes, the long-term prognosis for renal recovery is favorable, with three-quarters of patients achieving complete recovery within one year [1]. Patients and families should be informed that recovery may take several months, particularly in severe stroke cases, but that adherence to conservative management is usually rewarded with good outcomes.

## Conclusions

This study demonstrates that stroke-associated AKI is largely a reversible acute injury, with three-quarters of patients achieving complete functional recovery without requiring dialysis. Although severe stroke patients universally experienced AKI, ultimate recovery rates were comparable across stroke types and severities, with stroke severity primarily affecting recovery kinetics (median 8.2 months for severe cases) rather than overall recovery potential. The absence of dialysis requirements over 12 months supports conservative management strategies focused on monitoring rather than aggressive intervention. These findings underscore the value of structured outpatient follow-up protocols and extended monitoring in resource-limited settings, allowing clinicians to counsel patients and families that long-term renal prognosis is generally favorable.
